# DIGITAL TWIN NEIGHBORHOODS FOR PRECISION POPULATION HEALTH: INITIAL RESULTS OF COMMUNITY CONVERSATIONS

**DOI:** 10.1093/geroni/igad104.0171

**Published:** 2023-12-21

**Authors:** Adam Perzynski, Kristen Berg, Jarrod Dalton

**Affiliations:** The MetroHealth System, Cleveland, Ohio, United States; The MetroHealth System, Cleveland, Ohio, United States; Cleveland Clinic, Cleveland, Ohio, United States

## Abstract

Neighborhood socioeconomic inequality leads to lower use of preventive services, increased incidence and severity of disease, reduced quality of life, and higher mortality across multiple age groups. To accelerate discovery and prioritize population health strategies, we introduced Digital Twin Neighborhoods (DTNs): digital replicas of real communities that include biological, social, and geographic data and algorithms. We implemented a series of community engagement studio sessions (CES) that aim to elicit and clarify community members’ preferences and concerns about DTNs in order to most inclusively and ethically align DTN investigators’ research with community priorities. More than 30 participants have attended quarterly sessions. At CES sessions, researchers summarize the DTN framework and then engage with questions from (i) community experts and (ii) scientific experts, and (iii) open dialogue to guide project decision-making. To date, salient community experts’ concerns include: potential harms of DTNs for real-life communities; data privacy; selection of spatial indicators; and consequences for vulnerable populations. Suggestions for making DTN work more community-resonant have included: data transparency directly with residents; reconciling data definitions with local meanings of health; recognizing biases in electronic health records; and conducting shared activities within neighborhoods. CES sessions revealed foci for future DTN analyses including: how social isolation and togetherness influence health; identification of spatially defined targets for remediating structural racism; analysis of health-promoting assets’ (e.g., green space, churches) differential benefits; and examining how resilience creates practical advantages amidst difficult socioenvironmental circumstances. Conversing with community members, we are now developing implementation plans for each of these areas.

